# Vision Screening in Older Adults Admitted with a Fragility Hip Fracture: A Healthcare Quality Improvement Report

**DOI:** 10.22599/bioj.331

**Published:** 2023-11-23

**Authors:** Aishah Baig, Alexander Foss, Opinder Sahota, Khosrow Sehat, Isabel Ash

**Affiliations:** 1 Nottingham University Hospitals NHS Trust, Nottingham, UK

**Keywords:** Falls, Fragility hip fracture, Vision screening

## Abstract

**Background:**

This healthcare quality improvement report focussed on the effectiveness of an orthoptic-led inpatient vision screening service at Nottingham University Hospitals for older adults admitted with a fragility hip fracture. The service was developed in response to national guidance, which recommended a multifactorial assessment, including a vision assessment for older adults presenting following a fall.

**Method:**

Vision screening was carried out by orthoptists on eligible patients ≥65 years of age admitted to the trauma and orthopaedic wards with a hip fracture. Retrospective data for patients screened between 2015–2019 were analysed, including: patient demographics; screening eligibility and outcome; ophthalmology referrals made; ophthalmology appointment attendance; and outcome.

**Results:**

Of the 3321 patients admitted with a hip fracture between 2015–2019, 2033 (61%) were eligible for vision screening and 1532 (75%) of these were screened. Furthermore, 784 (51%) of the patients screened had an ocular abnormality requiring an ophthalmology referral, or a sight test at an optician. Only 144 of the 383 (38%) who required an ophthalmology referral via the GP were successfully referred, and only 107 of the 186 (58%) patients who were given appointments attended them. Additionally, 98 of 107 had pathology, with cataracts the most common finding (51%), and 61 of 98 (62%) patients had treatable vision impairment.

**Conclusions:**

We found a large proportion of fragility hip fracture patients with impaired vision, much of which was treatable and could be detected effectively with orthoptic-led bedside screening. The most common eye problem in those referred to ophthalmology was cataracts. An internal referral pathway to ophthalmology is proposed. There is a need to investigate reasons for disengagement with eye care services in this population.

## Introduction

Falls occur at least once a year in approximately 30% of the population aged 65 and over, and in 50% of those aged 80 and over (Campbell 1996; Masud & Morris 2001; NICE 2013). Approximately 5% of these falls lead to a fragility fracture (NICE 2018; Svedbom et al. 2013), which have significant and extensive consequences for the patient, health and care services. Hip fractures alone cost the UK National Health Service (NHS) an estimated £2 billion (of a total £4.4 billion) spent on fragility fractures each year (NICE 2018; Svedbom et al. 2013).

Impaired vision is one of many risk factors associated with falls and hip fractures (Cox et al. 2005a; Cox et al. 2005b; Felson et al. 1989; Mehta 2020; Mehta et al. 2022; Squirrell et al. 2005). The assessment and intervention for vision impairment has been included in national and international guidance for falls prevention (NICE 2013; Montero-Odasso et al. 2022). The National Institute for Health and Care Excellence (NICE) recommend a multifactorial risk assessment and intervention for patients who present to a healthcare setting following a fall or report recurrent falls or balance problems in the past 12 months. This includes an assessment and intervention for vision impairment (NICE 2013).

Despite the national guidance, implementation across the UK is inconsistent. A scoping study on falls services in the UK, 96% of which were hospital-based, found great variability in service provisions for vision assessments. Vision assessments were conducted in 58% of services, of which only 13% included a formal assessment. There was also a discrepancy between assessment and intervention, with only 35% of services intervening via direct action or onward referral (Lamb et al. 2008).

The Royal College of Physicians (RCP) published the ‘Report of the national audit of falls and bone health in older people’ in 2011. Overall, 42% of services included a formal vision assessment as part of the falls risk assessment, which was an improvement from the study by Lamb et al. (2008). Formal vision assessments were only reported in 10% of non-hip fracture and 17% of hip fracture patients (RCP 2011).

One barrier to implementing this service may be the ambiguity regarding the requirements of the recommended vision assessment, for example: which visual functions are to be assessed and how; who would perform the assessment; when and where the assessment should take place; and pathways to intervention for detected vision impairment. In order to tackle this, for the purposes of assessing vision in all older inpatients to prevent inpatient falls, the RCP introduced the ‘Look Out! Bedside vision check for falls prevention’ assessment tool in 2017 to enable any suitably trained health professional to conduct the vision assessment in an inpatient setting. This was developed collaboratively with the British and Irish Orthoptic Society, the College of Optometrists, the Royal College of Ophthalmologists, the Royal College of Nursing and NHS Improvement (RCP 2017). Even with this tool, the latest RCP ‘National Audit of Inpatient Falls annual report’ found that only 52% of inpatient femoral fractures had a vision assessment prior to the fall (RCP 2022).

The Trauma and Orthopaedics (T&O) and Ophthalmology departments at Nottingham University Hospitals Trust (NUHT) developed an inpatient vision screening service in 2013. This service targeted older adults admitted with a fragility hip fracture, which is among the most serious injuries following a fall (Keene, Parker & Pryor 1993) and one of the most devastating fractures in older age (Cummings et al. 1985)—with older adults considered a high risk population for repeat falls and fractures (Kanis et al. 2004; Klotzbuecher et al. 2000; Langridge et al. 2007; Llopis-Cardona et al. 2022; Rubenstein et al. 2001). The service was designed based on that by Squirrell et al. (2005) for older adults who have specifically suffered a hip fracture following a fall. These authors found that 52/89 of their cohort had visual acuity of 6/18 or worse in at least one eye; 40 patients had remedial vision impairment; and this could be detected effectively with a bedside vision assessment conducted by a suitably trained member of the multidisciplinary team.

In order to determine the effectiveness of this service at NUHT, a healthcare quality improvement project was conducted.

## Methods

Bedside screening was carried out on the T&O wards by an orthoptist two mornings per week. All patients ≥65 years of age admitted with a hip fracture were screened, excluding those already registered as sight impaired or severely sight impaired, those with cognitive impairment indicated by an abbreviated mental test score of <6/10, those under the macula service or with a contagious disease, on end-of-life care, or those not cared for on T&O wards due to the presence of serious other medical conditions.

The screening assessment comprised: patient history; best corrected uniocular distance visual acuity using the Keeler Crowded LogMAR test at three metres; near and distance cover test; ocular motility testing if indicated by cover test findings; pupil check; red reflex test; and binocular visual fields to confrontation. The outcomes of the screening are detailed in a flowchart in Appendix 1. The red reflex test was used to aid in the triage of the patient to a cataract specialist if a media opacity was suspected but was not used to determine screening outcome alone. If a relative afferent pupillary defect was found on the pupil assessment, advice was to be sought from an on-call ophthalmologist. The vision screening outcome was documented in the patient’s medical records and shared with the patient and their General Practitioner (GP) by letter.

As part of the healthcare quality improvement project, retrospective data was analysed for patients screened between 1 January 2015 and 31 December 2019. Data was analysed for patients seen prior to the height of the COVID-19 pandemic due to the suspension of the service in part of 2020 and 2021. Therefore, the data presented here reflects the usual running of the service.

Hospital systems and records were used to identify whether a referral to ophthalmology took place, appointment attendance, diagnoses and appointment outcomes.

### Ethical Approval

As a healthcare quality improvement project, ethical approval was not required, but approval was obtained by the clinical effectiveness team at NUHT [Audit reference number: 22–146C].

## Results

**Figure 1:**
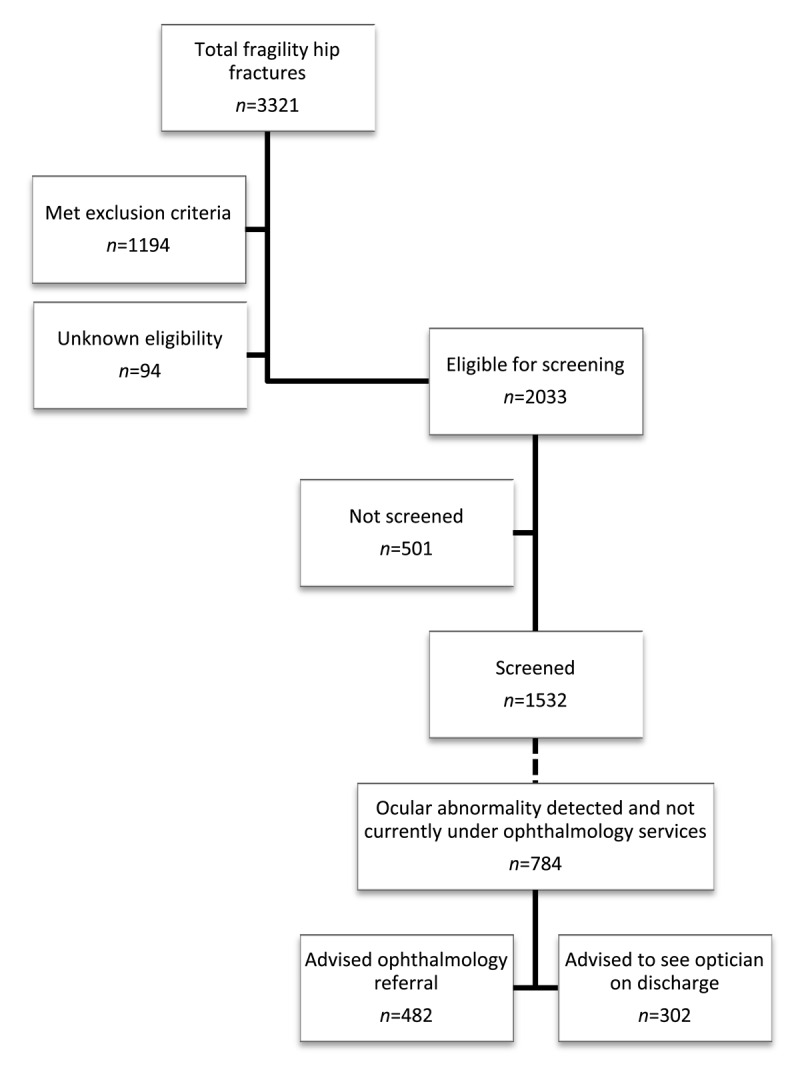
Eligibility for screening.

During the five-year period between 1 January 2015 and 31 December 2019 at NUHT, 3321 patients aged ≥65 years admitted with a fragility hip fracture were identified. This included 2354 female (71%) and 967 male (29%) patients with a mean age of 81.2 ± 11 and an age range of 65–102 years old. Of these, 2033 patients (61%) were eligible for vision screening after the exclusion criteria was applied. This included 1432 female (70%) and 601 male (30%) patients with a mean age of 82.3 ± 7.7 and an age range of 65–102 years old. Figure 1 shows the eligibility of patients and the number screened.

Exclusion criteria was met in 1194 cases (36%), and eligibility was unknown before the patient was discharged in 94 cases (2.6%). This included 922 female (72%) and 366 male (28%) patients with a mean age of 81.3 ± 11 and an age range of 65–102 years old. Table 1 records the number of patients meeting the exclusion criteria and the reasons for unknown eligibility in patients.

**Table 1:** Number of patients meeting exclusion criteria and reason for unknown eligibility.


EXCLUSION CRITERIA	*n*	% OF THOSE EXCLUDED	% OF TOTAL HIP FRACTURES

AMT <6	899	75%	27%

Not on T&O wards	73	6.1%	2.2%

Registered sight impaired/severely sight impaired	75	6.3%	2.3%

Current patient of the macular clinic	87	7.3%	2.6%

On end-of-life care	11	0.9%	0.3%

Deceased before eligibility assessed	49	4.1%	1.5%

Total no.	1194		

**REASON FOR UNKNOWN ELIGIBILITY**	***n***	**% OF THOSE WHERE ELIGIBILITY WAS UNKNOWN**	**% OF TOTAL HIP FRACTURES**

Discharged before AMT available to determine eligibility	26	28%	0.8%

Patient moved ward before able to determine eligibility	7	7.4%	0.2%

Screening session short-staffed	61	65%	1.8%

Total no.	94		


The most common reason for patients meeting exclusion criteria was having an AMT score below 6. This comprised 75% of those who met the exclusion criteria and 27% of the total population of older adults with a hip fracture. The most common reason for the inability to ascertain patient eligibility was screening sessions being short-staffed. This accounted for 65% of cases where eligibility was unknown. Screening staff were either absent, or screening sessions had to be cancelled to cover other short-staffed clinics.

In total, 1532 patients received a vision screening assessment; 1064 (69%) were female and 468 (31%) male with a mean age of 81.2 ± 11 and an age range of 65–102 years old. This constituted 75% of those eligible for screening. Whilst there were 501 eligible patients, 25% of those eligible were not screened. This included 368 female (73%) and 133 male (27%) patients with a mean age of 81.2 ± 11 and an age range of 65–100 years old. The various reasons for patients not being screened are shown in Table 2.

**Table 2:** Reasons for eligible patients not being screened.


REASON	*n*	% OF ELIGIBLE NOT SCREENED	% OF TOTAL ELIGIBLE

Patient declined screening	386	77%	19%

Discharged before being able to screen. This includes screening sessions being short-staffed and patients being discharged early	55	11%	2.7%

Unable to complete screening due to cognition despite AMT >6	39	7.8%	2%

Deceased after proving eligible	5	1%	0.3%

Patient had contagious disease	9	1.8%	0.4%

Moved ward after proving eligible	6	1.2%	0.3%

Language barrier	1	0.2%	0.05%

Total no.	501		


The most common reason for eligible patients not being screened was that they declined screening, which represents 77% of the reasons for eligible patients not being screened and 19% of the total number of those eligible for screening. Staff and patient education on the importance of screening reduced the decline rate within the six-month period between July and December 2022 from 19% to 4%.

Table 3 breaks down the patient outcomes following screening. Overall, 784 of 1532 (51%) screened patients were found to have an ocular abnormality and were not currently under ophthalmology services. Of these, 521 were female (66%), and 263 were male (34%) with a mean age of 83.2 ± 7.8, and a range of 65–102 years old. This included 521 of the 1064 (49%) females screened and 263 of the 468 (56%) males screened. These patients were advised further investigation by an optician on discharge or a referral to ophthalmology.

**Table 3:** Vision screening patient outcomes.


OUTCOME/ADVICE	*n*	% OF TOTAL SCREENED

For current ophthalmology patients		

Keep current appointment schedule	152	9.9%

Request expedited appointment	9	0.6%

For non-current ophthalmology patients		

Eye test at optician advised on discharge	302	20%

Regular eye tests advised at local optician	587	38%

Routine ophthalmology referral via the GP	375	24%

Urgent ophthalmology referral via the GP	8	0.5%

Urgent internal referral/referral to the macular clinic	51	3.3%

Patient declined referral	48	3.1%

Total no.	1532	


Patients advised to see a local optician on discharge constituted 302 of 784 (39%). The remaining 482 patients (61%) were advised a referral to an ophthalmology department; 383 required a referral via their GP, while 51 were internally referred due to a suspected serious/acute visual problem. Further, 48 refused a referral.

Of the 383 patients advised referral via the GP to the ophthalmology department, only 144 (38%) referrals were received by NUHT.

**Figure 2:**
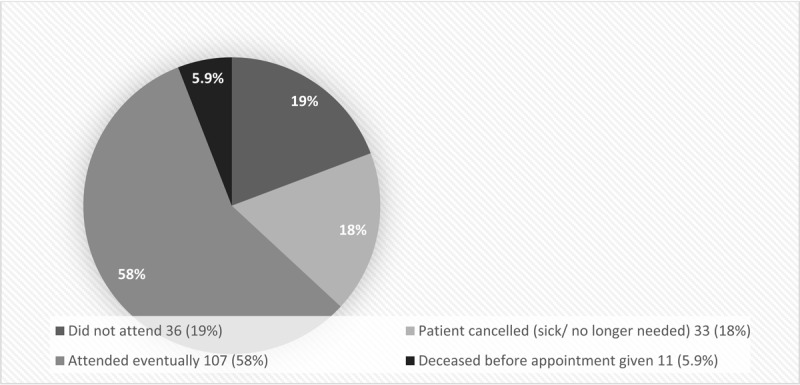
Attendance to outpatient appointments given.

In addition to the 144 patients referred via their GP to NUHT, a further 42 of 51 internal referrals were successful. Nine internal referrals were unsuccessful for unknown reasons. Figure 2 shows the attendance rate to outpatient appointments given. Those who eventually attended their appointment consisted of 107 of 186 (58%) patients; 36 (19%) did not attend, 33 (18%) cancelled and did not rebook, and 11 (5.9%) died after being given an appointment. Those considered part of the ‘did not attend’ group were still alive at the time of the non-attendance.

Ninety-eight of 107 (92%) patients who attended their appointments tested positive for pathology. Their diagnoses are listed in Table 4. Some patients had more than one diagnosis. Some findings, as specified in the table, include historic ocular defects that the patient had forgotten and were not readily available in the NUHT records.

**Table 4:** Diagnoses of patients referred and attending appointments at NUHT.


DIAGNOSES	NUMBER OF PATIENTS

Bilateral/unilateral cataract	50

Bilateral/unilateral posterior capsule opacification	15

Bilateral/unilateral dry age-related macular degeneration	19

Bilateral/unilateral wet age-related macular degeneration	1

Ocular motility abnormality	14

Likely amblyopia *(not previously documented or known by patient)*	5

Bilateral epiretinal membrane	1

Refractive error (myopia induced by cataract)	2

(suspect) Glaucoma/Ocular hypertension	6

Non-arteritic anterior ischaemic optic neuropathy	1

Signifiant dry eye	1

Pseudovitelliform lesion	1

Quadrantanopia	1

Iatrogenic dilated pupil	2

Posterior vitreous detachment	1

Upper lid basal cell carcinoma	1

Old retinal detachment	1

Bilateral aphakia (treated congenital cataracts) and nystagmus	1

Unexplained monocular diplopia	1

Involutional ptosis	1

Ptosis secondary to recent repeat botox treatment for hemifacial spasms	1


Cataracts were the most common finding (*n* = 50), followed by bilateral/unilateral dry AMD (*n* = 19) and bilateral/unilateral posterior capsule opacification (*n* = 15). Ocular motility abnormalities closely followed (*n =* 14). The leading primary causes of reduced visual acuity were cataract/PCO in 54 individuals, followed by AMD in 9.

**Figure 3:**
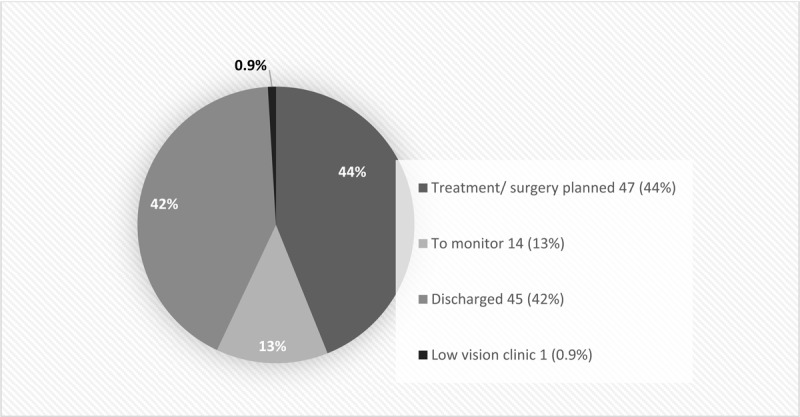
Outcome of attendance to outpatient appointments.

Overall, 61 of 98 (62%) patients with a diagnosis were offered treatment. The outcome of attendance to appointments in ophthalmology outpatients is shown in Figure 3. Forty-seven patients consented to treatment, which was 44% of the 107 who attended their appointment, 48% of those with pathology and 77% of those offered treatment. Six of these patients later did not attend, cancelled or died before treatment could proceed; 14 of 107 (13%) were monitored; 45 (42%) were discharged, 14 of which were discharged as they declined intervention, including nine who were offered cataract surgery. Referral to a low vision clinic was made for one (0.9%) patient. Treatment given consisted of cataract surgery (*n* = 21), YAG laser treatment for PCO (*n* = 9), YAG laser for peripheral iridotomy (*n* = 1), strabismus surgery (*n* = 1), Fresnel prisms/occlusive patch or orthoptic exercises (*n* = 6), intravitreal anti-VEGF therapy (*n* = 1) and topical medication (eye drops) (*n* = 4). Some patients had more than one form of treatment.

Of all the patients referred urgently to the macular clinic and seen, nine had AMD, including only one patient with neovascular AMD requiring intravitreal therapy, a further nine had cataracts/PCO and eight had other conditions.

The red reflex test produced low sensitivity and specificity for the detection of media opacities: 56% and 62%, respectively.

The patients’ reported last attendance at their local optician was analysed for those screened in 2019. This information was not recorded in 28 patients; therefore out of 300 records, 18 (6%) patients were unsure, four (1.3%) patients had never been to an optician to their knowledge, 230 (76.7%) had reported going to the optician within two years, 18 (6%) had been within two to three years and 30 (10%) had been more than three years ago. For those who specifically failed the screening, the patients’ last reported attendance at their local optician was not recorded for nine patients. Therefore, out of 125 records, eight (6.4%) patients were unsure, and three (2.4%) patients had never been to an optician to their knowledge. In total, 82 (65.6%) had reported going to the optician within two years, 11 (8.8%) had been within two to three years, and 21 (16.8%) had been more than three years ago.

## Discussion

This work evaluated the current vision screening service for older adults admitted with a hip fracture at NUHT between 2015 and 2019. At least half of patients screened over this five-year period had an ocular abnormality on screening and were not currently under ophthalmology services. These patients were either advised to see a local optician on discharge, if visual acuity improved with pinhole, indicating potential uncorrected refractive error, or were advised an onward referral to ophthalmology for further investigation of any other possible defect. Of those referred, 62% had a treatable condition.

Seven hundred and eighty-four patients were advised further investigation following screening and were not currently under ophthalmology. Uncorrected refractive error, suspected due to pinhole test results, but not confirmed by refraction, was one of the most common findings (302/784). Cataracts/PCO (65/98) and AMD (19/98) were the most common findings in those referred and seen in the NUHT ophthalmology department. It is also important to note that a further 4.9% (162/3321) of the total number of older patients with a fragility hip fracture in this report were already registered as sight impaired or severely sight impaired, or a current patient of the macula clinic and thus not eligible for screening.

Similarly, the literature demonstrates that the most common eye conditions found in this population with fragility hip fractures are: cataracts (Cox et al. 2005a; Cox et al. 2005b; Felson et al. 1989; Squirrell et al. 2005) and uncorrected refractive error (Cox et al. 2005a; Cox et al. 2005b; Squirrell et al. 2005), followed by AMD (Cox et al. 2005a; Cox et al. 2005b; Squirrell et al. 2005). In comparison, subjectively reported falls in a recent sample of older ophthalmology outpatients have been found to be most associated with glaucoma, followed by cataracts (Mehta, Knowles & Wilson 2021).

Squirrell and colleagues (2005) found that 58% of their cohort had impaired visual acuity of 6/18 or worse in at least one eye. The main causes being cataracts or related pathology, uncorrected refractive error and AMD. In a larger study, Cox et al. (2005b) found that 46% of their cohort had impaired vision, although this was determined by binocular visual acuity of 6/18 or worse, which potentially underestimates their findings. Vision impairment by their definition was principally due to cataracts, followed by AMD and uncorrected refractive error. This report focussed on the outcome of screening, which was not reliant on visual acuity result alone; therefore, it is not possible to directly compare results to these studies. We also demonstrated a larger proportion of this population with ocular abnormalities compared to the literature, which may be due to the larger sample size and the fact that ocular abnormalities other than reduced visual acuity were included.

This screening service detects ocular motility abnormalities as well as reduced visual acuity. Strabismus or ocular motility defects with diplopia were the primary reasons for referring 57 patients to ophthalmology. Ocular motility abnormalities may impact the risk of falling and hip fractures (Cummings et al. 1995; Felson et al. 1989; Ivers et al. 2000; Mehta 2020; Mehta et al. 2022) by impeding stability through reduced depth perception (Buckley et al. 2010; Lord & Menz 2000; Swenor et al. 2015; Vale, Buckley & Elliott 2008). Stereopsis and/or a brief ocular motility assessment should be recommended as part of any vision screening assessment for this population.

Of all the patients referred urgently to the macular clinic and seen, only one patient had neovascular AMD. Therefore, the criteria for referral has been updated to include the disclosure of symptoms consistent with macular oedema, as well as vision less than 0.300 (6/12), worsening with pinhole by at least one line, see Appendix 1.

The red reflex test was originally part of the screening assessment to help in the detection of cataracts or media opacities aiding the triage of referrals. However, our data have found the sensitivity and specificity of the test to be low: 56% and 62%, respectively. This low level of sensitivity is comparable or even higher than that reported in the findings of Squirrell et al. (2005), who found the red reflex test to have 40% sensitivity for detecting cataracts. However, their findings revealed a much higher specificity of 96%. Our results may be of limited significance, as it is based on a limited number of cases from the whole population. The sensitivity of the test may be impacted by the lighting conditions on wards, which are not always dim enough for an accurate bedside red reflex test, affecting the detection of less dense opacities. Due to the low sensitivity and specificity found, the red reflex test has been removed from the current screening protocol.

The majority of this population have impaired vision that can be treated with cataract surgery or glasses. These are likely cost-effective interventions for the prevention of falls and fractures. However, this has not yet been substantiated, and available research does not yet demonstrate a strong effect on falls and fracture incidence, likely due to the multifactorial nature of falls (Dhital, Pey & Stanford 2010; Gutiérrez-Robledo et al. 2021; Sach et al. 2007).

One randomised controlled trial (RCT) surprisingly found that correcting refractive error increased the risk of falls (Cummings et al. 2007). It was speculated that this may be due to an increase in physical activity following an improvement in vision and false confidence (Dhital et al. 2010). On the other hand, it appeared that participants were more likely to fall if they required a large increase in refractive correction, which is in keeping with other studies analysing falls and refractive error changes post-cataract surgery (Elliott, Black & Wood 2019; Keay & Palagyi 2018; Palagyi et al. 2017). Hence, we advocate for the promotion of regular sight tests to reduce the likelihood of requiring sudden large refractive changes, which may predispose patients to falls.

Haran and colleagues (2010) conducted an RCT involving an intervention of single-vision distance glasses for walking or standing activities outside of the home in long-term wearers of multifocal glasses. Participants in the intervention group who had a higher self-reported baseline outdoor activity level experienced a significant reduction in falls, whilst those less active experienced an increase in falls. Activity levels were found to be unchanged, therefore the authors suggested that more frail individuals may have found added difficulty in adapting to single-vision lenses for outdoor activities that they find difficult already. Mehta, Knowles and Wilson (2021) also found that the wearing of single-vision glasses compared to multifocal glasses was also significantly associated with the prevalence of falls in an ophthalmic outpatient department. This evidence may reinforce the need for multicomponent intervention in this population, whereby in order to benefit from improvements in vision, strength and balance also need to be actively optimised.

A recent meta-analysis found that timely first-eye cataract surgery significantly reduced the risk of recurrent falls, but there was no statistically significant impact following second eye surgery (Gutiérrez-Robledo et al. 2021). This meta-analysis included two RCTs on expedited surgery vs typical waiting times and six quasi-experimental studies comparing the incidence of falls before and after cataract surgery. Only three studies discussed second eye surgery.

Regarding the two RCTs, a significantly reduced rate of subjectively reported falls was associated with expedited first eye cataract surgery (Harwood et al. 2005). The same authors found no statistical significance, but a trend towards a decrease in reported falls after second eye surgery (Foss et al. 2006). Following this meta-analysis, Keay et al. (2022) conducted a large prospective observational study, which found a significant reduction in reported falls following first eye surgery and more so after second eye surgery. This is in keeping with a smaller study by Feng et al. (2018).

There is a need to detect both correctable vision impairment and that which cannot be treated. Increasing awareness of vision impairment, prompt referral to low vision services and working with Occupational Therapists to improve the home environment may help reduce falls risk and improve quality of life.

The literature and results of this healthcare quality improvement project support a screening assessment in this population, which both detects vision impairment and distinguishes between advising a referral to a local optician for refractive correction and a timely referral to ophthalmology for management of other conditions such as cataracts.

Hospital records for screened patients referred to the NUHT ophthalmology department revealed that 92% had genuine pathology, indicating accuracy with current screening methods. However, this was determined for a limited number of patients, as 62% of the patients advised referrals were not referred, and only 58% of referred patients attended their appointment.

In order to reduce the number of missed referrals, a trial of a direct referral pathway from the orthoptist screening to a hospital optometrist-led clinic is proposed. Presently, organisational arrangements in the English NHS for this service necessitate the referral of patients by their GP to the ophthalmology department for non-urgent conditions, which may present a barrier due to the current strain on GP resources. An internal referral pathway would introduce better efficiency and improved efficacy.

Engagement with eye care services may be suboptimal in this population, including secondary care, which is free of charge in the NHS. Half of those screened in our population required onward referral, 39% of which were suspected of uncorrected refractive error, suggesting inadequate contact with opticians. Although with the increase in domiciliary sight test availability, an increase in test uptake and decrease in vision impairment due to uncorrected refractive error would be expected. For those screened in 2019, 76.7% of patients reported seeing an optician within the previous two years. It is a slight increase from 65% reported by Cox and colleagues in 2005, likely reflecting initiatives by organisations such as the Royal College of Physicians, the National Falls Prevention Coordination Group, the College of Optometrists and optical retail companies to promote regular eye tests for the public, offer domiciliary eye tests and raise awareness of the association between poor vision and falls. Furthermore, 37% of patients referred to ophthalmology from screening did not attend or cancelled their appointment. The value of screening may be diminished by the lack of engagement with follow-up care. Qualitative research is needed seeking the views of this population and their carers on the priority of eye health and barriers to engagement with eye services.

The inpatient vision screening service discussed here, based on that by Squirrell et al. (2005), presents a practical model for detecting vision problems in a high-risk population for further falls and fractures (Kanis et al. 2004; Klotzbuecher et al. 2000; Langridge et al. 2007; Llopis-Cardona et al. 2022; Rubenstein, Powers & MacLean 2001). This model utilises the multidisciplinary team, which is shown to be a cost-effective strategy in hip fracture prevention (Sanders et al. 2021). Rather than deferring all patients to a local optician on discharge, an initial inpatient vision assessment, performed as part of a multifactorial risk assessment coordinated by the hospital care provider, may be preferable in many ways. Firstly, for the patient, an assessment within this acute context specifically as part of a falls prevention programme may raise the importance of vision as an important and manageable risk factor for falls. It also removes any transport difficulties, related time and cost implications, although this may not apply for patients who agree to a domiciliary sight test by an optician. However, it removes the need for the patient to remember to arrange the initial sight test and may inform rehabilitative and discharge planning.

The vision screening assessment described here quantifies visual acuity accurately enough to be able to distinguish which patients may require a timely referral to an ophthalmology department for potential surgical/medical intervention and which may require (updated) glasses by their local optician. It also detects other visual risk factors for falls and fragility hip fractures, such as visual field and binocular vision defects. In contrast, more recently developed vision screening tools, such as the Royal College of Physicians ‘Look Out!’ Bedside vision check for falls prevention’ and the Thomas Pocklington Trust ‘Eyes Right Toolkit’ are intended for the detection of gross vision defects, optimise the individual’s immediate environment and do not have a well-defined management pathway for those requiring timely follow-up care in an ophthalmology department. Mehta et al. (2022) recently showed that the two visual risk factors most associated with falls were stereopsis and contrast sensitivity. The best portable, reliable methods to test these additional visual functions—and how results would determine screening outcomes and management—should be considered prior to incorporation into a vision screening assessment specific to falls.

This vision screening model may be adapted to capture the wider population of older adults hospitalised following a fall or fragility fracture in order to achieve national standards, which may currently not be met. Orthoptists are well-placed to lead this vision screening service and train other health care professionals to conduct screening in a way similar to those done in school-aged vision screenings or vision screenings in stroke patients. An orthoptic-led service may aid in the management, coordination and implementation of the service to help meet national standards.

To expand our service, eligible hip fracture patients cared for on non-T&O wards will be included for screening in the future. Staff and patient education on the importance of screening has also proven beneficial in reducing the decline rate. Eligible patients who cannot be screened for any reason will be sent letters reinforcing the importance of regular sight tests.

A limitation of this healthcare quality improvement project was the reliance on the accurate input of data by screeners onto the spreadsheet used for part of the data collection. This data included patient demographics, screening eligibility and screening outcome. Data is only presented for one hospital, Trust; therefore, the data may not reflect the wider population of older adults with a fragility hip fracture across the UK. However, the results are in keeping with previous literature from other areas of the UK (Cox et al. 2005b; Squirrell et al. 2005).

Patients who were advised to see an optician were not followed-up, so it is not possible to confirm the number of patients who followed this advice (seeing an optician) and had confirmed uncorrected refractive error. A study looking at a more recent sample of screened patients would be beneficial, which could also investigate compliance with prescribed glasses.

## Conclusion

In summary, a large proportion of older adults sustaining a fragility hip fracture have impaired vision, much of which is easily treatable and can be detected effectively with orthoptic-led bedside vision screening. This screening model could also be adapted for use in other patients hospitalised following a fall. Screening should distinguish between patients requiring timely referrals to ophthalmology and patients requiring a sight test with a community optician. Engagement with eye care services is lacking in this older population, and the reasons for this need to be further explored and addressed.

## Figures and Tables

**Appendix 1:**
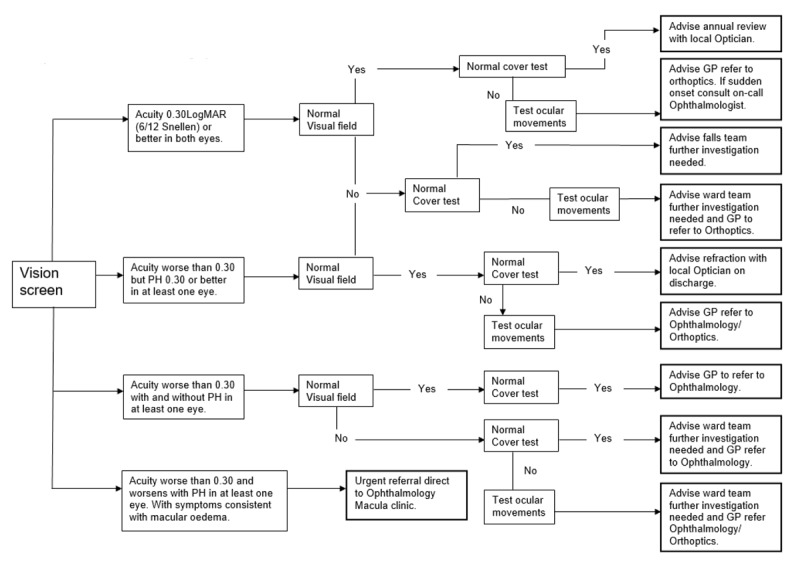
Vision screening outcomes.
